# Perinatal outcomes and predictors of placental abruption: a retrospective study in an Ethiopian tertiary care center

**DOI:** 10.3389/fpubh.2024.1453117

**Published:** 2025-01-07

**Authors:** Mesfin Tadese, Gebresenbet Getachew, Tirusew Nigussie Kebede, Toyba Ebrahim Yesuf, Saba Desta Tessema, Wogene Asefa Damesa, Gebeyehu Shumet Solomon

**Affiliations:** ^1^Department of Midwifery, School of Nursing and Midwifery, Asrat Woldeyes Health Science Campus, Debre Berhan University, Debre Berhan, Ethiopia; ^2^Department of Medicine, Obstetrician and Gynecologist, College of Medicine and Health Science, University of Gondar, Gondar, Ethiopia; ^3^Department of Medicine, Obstetrician and Gynecologist, Abebech Gobena Mothers and Childrens Health Hospital, Addis Ababa, Ethiopia; ^4^Department of Epidemiology, St. Peter Specialized Hospital, Addis Ababa, Ethiopia

**Keywords:** placental abruption, adverse perinatal outcome, factors, Ethiopia, antepartum hemorrhage

## Abstract

**Background:**

Placental abruption is a critical obstetric condition characterized by the premature separation of the placenta from the uterus, leading to severe maternal and fetal complications. In Ethiopia, the maternal and perinatal morbidity and mortality rates are alarmingly high, and placental abruption significantly contributes to these adverse outcomes. Despite its severity, there is a lack of comprehensive data on the burden, risk factors, and outcomes associated with placental abruption in the Ethiopian context. Thus, the study aimed to investigate the adverse perinatal outcomes of placental abruption and the factors associated with these outcomes among pregnant women admitted to the University of Gondar Comprehensive Specialized Hospital in Ethiopia.

**Method:**

An institution-based retrospective cross-sectional study was conducted among 367 pregnant women who were admitted and managed for placental abruption from January 1, 2021, to January 1, 2023, at the University of Gondar Comprehensive Specialized Hospital. A simple random sample method was employed to choose the medical records. Data was collected using a checklist prepared with the KOBO collect tool and then exported to SPSS version 25.0. Variables having a *p*-value of less than 0.05 were deemed significant in the multivariable logistic regression analysis that was done. The adjusted odds ratio with a 95% confidence interval was reported.

**Result:**

The prevalence of adverse perinatal outcome of placental abruption was 39.2, 95% CI: 34.3–44.1. The most common adverse outcomes were prematurity (25.6%), low birth weight (25.6%), and NICU admission (13.9%). Severe placental abruption [AOR (CI) = 8.82 (4.48–17.31)] and abruption at preterm gestation [AOR (CI) = 18.71 (9.59–36.42)] were significant predictors of adverse perinatal outcomes.

**Conclusion:**

The adverse perinatal outcomes of pregnancies complicated by placental abruption in this study were higher compared to other studies in Ethiopia. The degree of placental abruption and gestational age at diagnosis were significant associates of adverse perinatal outcomes. The study highlights the critical need for patient-centered counseling on antenatal bleeding to encourage early healthcare-seeking behavior, close follow up for those undergoing expectant management and the early detection and management of placental abruption to improve perinatal outcomes.

## Background

Placental abruption, also known as abruption placentae, occurs when bleeding at the decidual-placental interface leads to partial or complete detachment of the placenta before the fetus is delivered. This condition is typically diagnosed in pregnancies beyond 28 weeks of gestation. Major clinical symptoms include vaginal bleeding and abdominal pain, which are often accompanied by hypertonic uterine contractions, uterine tenderness, and a non-reassuring fetal heart rate pattern (1). Abruption occurs in approximately 0.6 to 1.2% of all pregnancies, with nearly half of cases happening at term gestations (1).

Abruption is a serious obstetric complication that poses significant risks for maternal and perinatal morbidity as well as perinatal mortality. The perinatal mortality rate is about 20 times higher in pregnancies with abruption compared to those without (12% versus 0.6%, respectively) (2). In Japan, placental abruption accounted for 9% of all stillbirths (3). It also led to preterm birth, perinatal asphyxia, intrauterine growth restriction, and low birth weight (1). The woman is at risk of severe hemorrhage, necessitating blood transfusions and potentially leading to complications such as hysterectomy, bleeding disorders like disseminated intravascular coagulopathy, and renal failure. These complications can lead to Sheehan syndrome or postpartum pituitary gland necrosis (4). In numerous countries, the occurrence of placental abruption has been on the rise despite advancements in obstetrical care and monitoring methods. This trend highlights a multifactorial etiology that remains poorly understood (5, 6). However, placental abruption is believed to result from a disruption of the maternal-fetal interface, specifically the decidual-placental junction. This disruption can be caused by various factors, including uteroplacental under perfusion, placental inflammation, or mechanical forces, leading to bleeding and subsequent separation of the placenta from the uterine wall (1). Identifying and managing the risk factors for placental abruption is crucial in mitigating these adverse outcomes.

The precise cause of placental abruption is unknown; however, various factors are associated with its occurrence. Placental abruption occurs in 40% of smokers, 14.1% of women with vasculoplacental disorders, and 42.2% of women with pre-eclampsia (7). Maternal age over 35 years, short umbilical cord, sudden decompression of the uterus, previous abruption, and trauma are also the strongest risk factors for abruption (4). Additionally, significant risk factors for placental abruption include frequent motorbike transportation, a history of infertility, and marginal cord insertion (8).

In Ethiopia, 30% of women with antepartum hemorrhage experienced adverse perinatal outcomes. Factors significantly associated with adverse maternal and perinatal outcomes include hemodynamic status, parity, antenatal care, duration of bleeding, gestational age, and the amount of vaginal bleeding (9). Uterine malformations, preterm premature rupture of membranes, and oligohydramnios significantly increased the risk of adverse perinatal outcomes of placental abruption (10). Additionally, maternal age of 20 years or younger, preeclampsia/eclampsia, and chronic hypertensive disorders during pregnancy were associated with adverse perinatal outcomes (11).

In Ethiopia, perinatal mortality rates remain high, and complications related to placental abruption contribute substantially to these adverse outcomes. Despite its severity, there is a lack of comprehensive data on the prevalence, risk factors, and outcomes associated with placental abruption in the Ethiopian healthcare settings. Thus, the study aims to examine the adverse perinatal outcomes associated with placental abruption and identify the factors contributing to these outcomes among pregnant women. Understanding these factors can inform clinical practices and policy interventions to improve maternal and neonatal health in Ethiopia.

## Method

### Study design, area, and period

From January 1, 2021, to January 1, 2023, an institution-based retrospective cross-sectional study was conducted at the University of Gondar Comprehensive Specialized Hospital (UOGCSH). UOGCSH is situated in Gondar, 741 kilometers away from Ethiopia’s capital, Addis Ababa. The hospital is a tertiary-level teaching and referral hospital in the Amhara region, which is one of the biggest and oldest medical schools in Ethiopia established as the Public Health College in 1954. UOGCSH provides various health services, including surgery, internal medicine, pathology, dermatology, obstetrics and gynecology, pediatric care, laboratory services, pharmacy, and physiotherapy. The labor and maternity ward is staffed by 8 gynecologic oncology subspecialists, 5 maternal-fetal medicine subspecialists, 4 urogynecology subspecialists, 7 subspecialist fellows, 5 obstetrics and gynecology specialists, 199 midwives, and 71 residents (20 first-year, 19 s-year, 23 third-year, and 13 fourth-year residents).

### Population and eligibility criteria

All randomly selected pregnant women admitted and managed for the diagnosis of placental abruption were the study population. Pregnant women with multiple gestation and lethal fetal congenital anomaly were excluded.

### Sample size and sampling techniques

The required sample size was determined using single population proportion formula for the first and second objective. Taking an assumption of power 80%, margin of error 5, 95% two-sided confidence level, and stillbirth in abruption 66% (12). After adding 10% missing records the final sample size was 380.

Computer-generated simple random sampling was used based on a sampling frame prepared by arranging medical record numbers in order from the maternity triage registration book. A total of 517 pregnant women were admitted with the diagnosis of placental abruption at UOGCSH from January 1, 2021, to January 1, 2023. Using simple random sampling, 380 pregnant women were selected, but 13 charts were missing, incomplete, or damaged.

### Covariates

Adverse perinatal outcome was the dependent variable. Socio demographic status included age, residency, marital status, and distance traveled to arrive the study hospital. Details of past and present obstetric factors included prior abortion, prior stillbirth, prior cesarean section, parity, gravidity, interpregnancy interval, ANC follow up, gestation age at diagnosis, polyhydramnios, oligohydramnios, preeclampsia, premature rupture of membrane, and gestational diabetes mellitus. Maternal characteristics included duration of bleeding, degree of placental abruption, blood pressure, anemia, onset of labor, and mode of delivery.

### Definition of terms

#### Placental abruption

Placental abruption was identified from participant medical records if there was a physician documented diagnosis of antepartum or intrapartum placental abruption, if it was noted as the indication for cesarean delivery, or if there was a discharge code indicating abruption.

#### Mild placental abruption

Clinically asymptomatic before delivery and typically detected by the existence of a retro-placental clotting and characterized by no vaginal bleeding to mild vaginal bleeding, slightly tender uterus, normal maternal BP and heart rate, no coagulopathy, and reassuring fetal heart rate (9).

#### Severe placental abruption

The presence of one or more of the following maternal or fetal complication in a patient diagnosed with placental abruption. Maternal: disseminated intravascular coagulation, hypovolemic shock, need for blood transfusion, hysterectomy, renal failure, and death. Fetal: non-reassuring fetal heart rate, intrauterine growth restriction, need for preterm birth, and death (13, 14).

#### Adverse perinatal outcome

The presence of at least one or more of the following: preterm birth (delivery before 37 completed weeks, but after 28 or more weeks of gestation), stillbirth (death of a fetus after 28 weeks of gestation, but before or during birth), low APGAR score (5^th^ minute APGAR is less than 7), low birth weight (<2,500 gm), IUGR (a birth weight of below 10^th^ percentile for gestational age and fetal sex), NICU admission, and need for resuscitation (15, 16).

#### Interpregnancy interval (IPI)

IPI is the period between the end of one pregnancy and the beginning of the next pregnancy. The IPI was categorized as short if <24 months, Optimal if 24 to <60 months, and long if more than or equal to 60 months (17).

#### Anemia

Anemia is a decrease in the concentration of erythrocytes or hemoglobin less than 11.0 g/dL / Hct 33%. Categorized into; Mild (10 to 10.9 g/dL), Moderate (7 to 9.9 g/dL), and Severe (7 g/dL) (18).

### Data collection tool and quality assurance

Data was collected using a structured checklist prepared with the KOBO collect tool. The checklist included demographic information (age, residence), obstetric history (parity, previous abruption), clinical presentation (gestational age at diagnosis, degree of abruption), and perinatal outcomes (birth weight, APGAR scores, NICU admission). A data extraction tool was developed after reviewing previous literature on the subject and validated by a reproductive health expert. A pre-test was conducted with 5% of the total sample size at Debre Berhan Comprehensive Specialized hospital and required modifications were considered. Three research midwife data collectors and two obstetrics and gynecology resident supervisors were included in the data collection process. Data collectors received 1 day of training on the study’s objectives, using the KOBO collect digital data extraction tool, accessing records, data handling, and maintaining participant confidentiality. The principal investigator checked the extracted data for completeness on a daily basis.

### Data processing and analysis

The data collected using KOBO collect was exported to SPSS version 25.0. Descriptive statistics were presented through frequency tables, graphs, and text. Binary logistic regression was employed to examine the association between dependent and independent variables. Multivariable logistic regression analysis was performed to identify independent predictors of adverse perinatal outcomes. Variables with *p* < 0.25 in the bivariate analysis were included in the multivariable model. The Hosmer-Lemeshow test was used to assess the goodness-of-fit of the model. Adjusted odds ratios (AOR) with 95% confidence intervals (CI) were calculated, and variables with *p* < 0.05 in the final model were considered statistically significant.

## Results

### Baseline characteristics

A total of 367 pregnant women diagnosed with placental abruption were included, representing 96.6% of the final sample size. The participants’ ages ranged from 17 to 40 years, with a mean age of 27.91 ± 5.5 years. Most participants (73.3%) were aged between 20 and 34, and 60% were urban residents (Table 1).

**Table 1:** Baseline characteristics of pregnant women admitted and managed for the diagnosis of placental abruption at University of Gondar Comprehensive Specialized Hospital (UOGCSH), Ethiopia, 2023.

Variables	Category	Frequency	Percent (%)
Age	<20	31	8.4%
	20–34	269	73.3%
	≥35	67	18.3%
Residence	Rural	148	40.3%
	Urban	219	59.7%
Marital status	Married	324	98.9%
	Others*	24	1.1%
Distance traveled to the UOGCSH	<15 min	206	56.1%
	15–30 min	14	3.8%
	≥30 min	147	40.1%

*Single, divorced, and widowed.

### Reproductive and obstetric characteristics

More than half (72%) of the women were multigravida, and 56.7% had a short interpregnancy interval. Additionally, 21.5% had previously experienced an abortion, and 4.4% had a history of stillbirth. The majority (93.7%) of participants had antenatal care (ANC) contact for the index pregnancy. Preeclampsia (6.8%), oligohydramnios (5.7%), and premature rupture of membrane (4.9%) were the commonest obstetrics complication identified during the index pregnancy (Table 2).

**Table 2:** Reproductive and obstetric characteristics of pregnant women admitted and managed for the diagnosis of placental abruption at UOGCSH, Ethiopia, 2023.

Variables	Category	Frequency	Percent (%)
Gravidity	Primigravida	103	28.1%
	Multigravida	264	71.9%
Parity	Nulliparous (0)	136	37.1%
	Multiparous (1 – 4)	178	48.5%
	Grand multiparous (≥5)	53	14.4%
Interpregnancy interval	Short (<24 months)	208	56.7%
	Optimal (24–59 months)	132	36.0%
	Long (≥ 60 months)	27	7.3%
History of abortion	No	288	78.5%
	Yes	79	21.5%
Number of abortion (*n* = 79)	One	68	86.1%
	Two or more	11	13.9%
History of stillbirth	No	351	95.6%
	Yes	16	4.4%
History of early neonatal death	No	340	92.6%
	Yes	27	7.4%
Previous cesarean section (CS)	No	347	94.6%
	Yes	20	5.4%
ANC follow up	No	23	6.3%
	Yes	344	93.7%
Preeclampsia	No	342	93.2%
	Yes	25	6.8%
Gestational diabetes mellitus	No	364	99.2%
	Yes	3	0.8%
Premature rupture of membrane	No	349	95.1%
	Yes	18	4.9%
Polyhydramnios	No	350	95.4%
	Yes	17	4.6%
Oligohydramnios	No	346	94.3%
	Yes	21	5.7%
HIV/AIDS	No	358	97.5%
	Yes	9	2.5%
RH status	Negative	27	7.4%
	Positive	340	92.6%

### Maternal condition at admission and delivery

Upon admission, 61% of the women reported bleeding for a duration of 12 h or less, and 58.6% presented at term. More than one-third (70.6%) did not have anemia at the time of admission. Additionally, among the mothers with placental abruption, 25.6% delivered preterm, and 34.1% underwent an emergency cesarean section (Table 3).

**Table 3:** Maternal conditions of pregnant women admitted and managed for the diagnosis of placental abruption at UOGCSH, Ethiopia, 2023.

Variables	Category	Frequency	Percent (%)
Duration of bleeding/symptoms of abruption	≤ 12 h	224	61.0%
	> 12 h	143	39.0%
Degree of placental abruption	Mild	184	50.1%
	Severe	183	49.9%
Gestational age at diagnosis	Preterm	140	38.1%
	Term	215	58.6%
	Post-term	12	3.3%
Systolic blood pressure (BP) at admission	<90 mmHg	4	1.1%
	90–139 mmHg	344	93.7%
	≥140 mmHg	19	5.2%
Diastolic BP at admission	<60 mmHg	7	1.9%
	60–89 mmHg	330	89.9%
	≥90 mmHg	30	8.2%
Anemia (Hemoglobin)	No anemia	259	70.6%
	Mild anemia	49	13.4%
	Moderate	45	12.2%
	Severe anemia	14	3.8%
Gestational age at delivery	Preterm	94	25.6%
	Term	259	70.6%
	Post-term	14	3.8%
Onset of labor	Elective CS	14	3.8%
	Induced	55	15.0%
	Spontaneous	298	81.2%
Mode of delivery	Elective CS	14	3.8%
	Emergency CS	125	34.1%
	Vaginal delivery	228	62.1%

### Prevalence of adverse perinatal outcome

**Figure 1:**
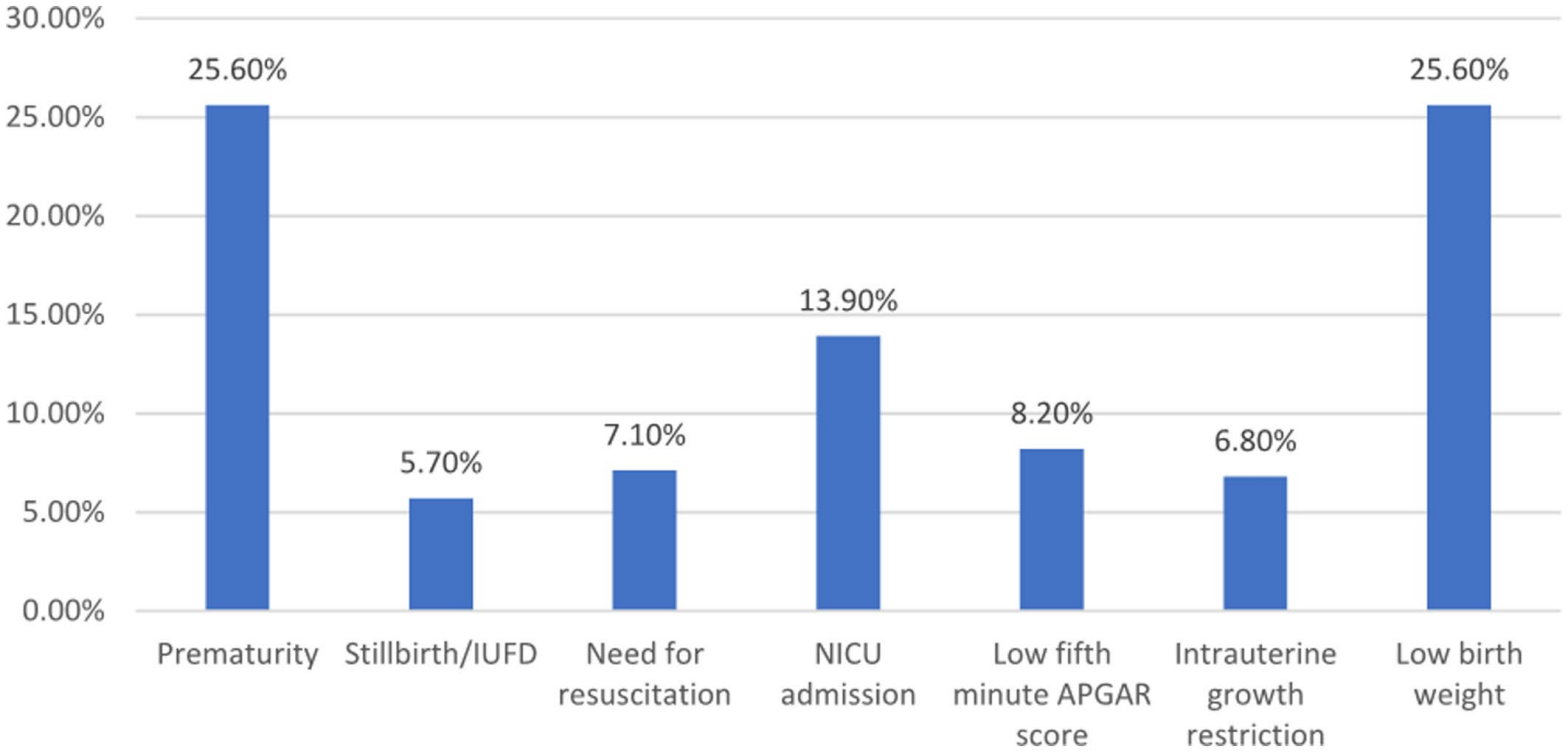
Adverse perinatal outcome among women admitted and managed for diagnosis of placental abruption at UOGCSII, Ethiopia, 2023. APGAR, Appearance, Pulse, Grimace, Activity, and Respiration; NICU, Neonatal intensive care unit; IUFD, Intrauterine fetal death.

In this study, 39.2% (*N* = 144) (95% CI: 34.3–44.1) of participants had one or more adverse perinatal outcome of placental abruption. As illustrated in Figure 1, the adverse perinatal outcomes varied in frequency. Prematurity and low birth weight were equally prevalent, each affecting 25.6% of cases. NICU admission was necessary for 13.9% of newborns, while 5.4% exhibited a low APGAR score at 5 min. Stillbirth occurred in 4.9% of cases, and 3.5% required neonatal resuscitation. These findings underscore the significant impact of placental abruption on neonatal health (Figure 1).

### Factors of adverse perinatal outcome

Bivariable and multivariable logistic regression analysis were conducted to identify factors associated with adverse perinatal outcomes. In the bivariable analysis, variables such as age, residence, parity, ANC follow-up, distance traveled, premature rupture of membranes, duration of bleeding, degree of placental abruption, systolic blood pressure, anemia, and gestational age at diagnosis had *p*-values <0.25 and were included in the multivariable model. The multivariable logistic regression analysis revealed that the degree of placental abruption and gestational age at diagnosis were significantly associated with adverse perinatal outcomes.

Women who developed severe placental abruption were eight times more likely to experience adverse perinatal outcomes compared to those with mild placental abruption [AOR (CI) = 8.82 (4.48–17.31)]. Additionally, women with placental abruption during preterm pregnancy were 18 times more likely to face adverse perinatal outcomes compared to those whose placental abruption occurred at term [AOR (CI) = 18.71 (9.59–36.42)] (Table 4).

**Table 4:** Factors associated with adverse perinatal outcome among women admitted and managed for diagnosis of placental abruption at UOGCSH, Ethiopia, 2023.

Variables	Adverse perinatal outcome		COR (95% CI)	AOR (95% CI)
	No	Yes		
Age				
<20	23	8	1	1
20–34	161	108	1.92 (0.83–4.47)	1.36 (0.38–4.86)
≥35	39	28	2.06 (0.81–5.28)	0.88 (0.18–4.33)
Residence				
Rural	70	78	2.58 (1.67–3.98)	0.95 (0.30–3.04)
Urba	153	66	1	1
Parity				
Primipara	85	51	1	1
Multipara	113	65	0.96 (0.60–1.52)	0.53 (0.25–1.14)
Grand multipara	25	28	1.87 (0.98–3.55)	0.64 (0.18–2.34)
ANC follow up				
No	8	15	3.13 (1.29–7.58)	3.31 (0.76–14.5)
Yes	215	129	1	1
Premature rupture of membrane (PROM)				
No	217	132	1	1
Yes	6	12	3.29 (1.21–8.97)	3.46 (0.73–16.3)
Duration of bleeding				
≤ 12 h.	142	82	1	1
> 12 h.	81	62	1.33 (0.86–2.03)	0.91 (0.46–1.78)
Degree of placental abruption				
Mild	161	23	1	1
Severe	62	121	13.6 (8.01–23.3)	8.82 (4.48–17.3)*
Systolic BP at admission				
<140 mmHg	214	134	1	1
≥140 mmHg	9	10	1.77 (0.70–4.48)	0.95 (0.24–3.80)
Anemia at admission				
No anemia	171	88	1	1
Anemia	52	56	1.46 (0.78–2.71)	1.24 (0.62–2.47)
Gestational age at diagnosis				
<37 weeks	28	112	24.4 (13.9–42.5)	18.7 (9.59–36.4)*
≥37 weeks	195	32	1	1
Distance of the hospital				
<15 min	148	58	1	1
15–30 min	7	7	2.55 (0.86–7.60)	2.05 (0.29–14.6)
≥30 min	68	79	2.96 (1.90–4.62)	1.76 (0.58–5.36)

BP, Blood pressure; * statistically significant at *p*-value < 0.05.

## Discussion

In this study, 39.2, 95% CI: 34.3–44.1 of pregnant women had developed at least one adverse perinatal outcome. The degree of placental abruption and gestational age at diagnosis were significantly associated with adverse perinatal outcomes.

The prevalence of adverse perinatal outcome among women admitted and managed for placental abruption was 39.2%. From these, 25.6% are premature, 25.6% are low birth weight (LBW), and 14% are referred to NICU. Similarly, in the United States, abruption was associated with an elevated risk of newborn resuscitation, asphyxia, respiratory distress syndrome, NICU admission, and stillbirth (19). It is also consistent with the study conducted in Nepal, where placental abruption is associated with preterm labor, low birth weight, and NICU admission (20). Chronic conditions such as thrombosis, inflammation, infection, and uteroplacental and decidual vasculopathy predispose to placental abruption. Placental hypoperfusion, impaired spiral artery remodeling, placental infarction, and shallow trophoblast invasion are the outcomes of these processes. These long-term changes heighten the risk of abruption and other placental-related complications, such as low birth weight (LBW), preterm birth, and fetal growth restriction (1).

However, this study has shown a higher prevalence of adverse perinatal outcomes than the study conducted at Southwest Ethiopia, where 30% of women with antepartum hemorrhage experienced adverse perinatal outcomes (9). This may be due to the ongoing conflict in the study area throughout the study period. This conflict could have caused transportation problems, preventing women from accessing maternity care when experiencing signs of vaginal bleeding. Consequently, women might present with major placental abruption and severe complications. Similarly, a systematic review and meta-analysis of studies in 12 conflict-zone countries reported an increased risk of small-for-gestational-age births, low 5th-minute APGAR scores, stillbirth, and perinatal mortality (21).

Additionally, our findings indicated a higher prevalence of placental abruption compared to a study conducted in Addis Ababa, where the prevalence was reported at 2.3% (22). This discrepancy may be attributed to the lower institutional delivery rate in the Amhara region, which stands at 54.2%, compared to 94.8% in Addis Ababa, according to the 2019 EDHS report. Similarly, 81.8% of pregnant women in Addis Ababa had four or more ANC visits, whereas only 50.8% in the Amhara region attended that many visits (23).

Preterm placental abruption at the time of admission increased the likelihood of adverse outcomes by 18 times compared to term abruption. Additionally, in this study, 38.1% of pregnant women presented before 37 weeks of gestation, and 25.6% delivered preterm. Patients with preterm abruption are typically managed expectantly if the fetomaternal condition is stable, with delivery planned for 37 to 38 weeks (13). However, these patients remain at risk of maternal or fetal complications due to progressive or recurrent placental separation, leading to relatively high neonatal morbidity and mortality. Partial abruption can suddenly and unpredictably progress to total abruption. Preventing preterm birth in pregnancies complicated by abruption is challenging. Consequently, urgent preterm operative delivery may be required without adequate preparation for neonatal care. Therefore, to reduce adverse perinatal outcome, it is recommended that most patients with acute abruption be delivered at 34 to 36 weeks of gestation, with continuous monitoring, optimal care, and thorough preparation.

Compared to women with mild placental abruption, those with severe abruption had an eight-fold increased risk of adverse perinatal outcomes. Similarly, a retrospective cohort research conducted in the United States found that women who experienced severe placental abruption had a 4.29-fold increased risk of serious maternal and neonatal complications, while women who experienced mild abruption had a 1.52-fold increased risk (14). Severe placental abruption might be characterized by heavy vaginal bleeding, total placental separation, tetanic uterus/ board-like consistency, hypofibrinogenemia and coagulopathy (4). This may aggravate the adverse obstetric and perinatal outcomes.

Our findings emphasize the critical need for vigilant monitoring of pregnancies complicated by placental abruption, particularly those presenting before term. The high rates of prematurity and low birth weight suggest that early detection and management of abruption may be crucial in improving perinatal outcomes. Furthermore, the increased risk associated with severe abruption highlights the importance of prompt recognition and intervention in these cases. These results may inform clinical protocols for the management of placental abruption, potentially including more frequent fetal monitoring and earlier consideration of delivery in cases of severe abruption.

### Limitation

This study has several limitations that should be considered when interpreting the results. First, as a retrospective study, it is subject to inherent biases, including potential inaccuracies in medical records and missing data. Second, the single-center design may limit the generalizability of our findings to other settings, particularly those with different resources or patient populations. Third, we were unable to assess long-term neonatal outcomes beyond the immediate perinatal period, which may underestimate the full impact of placental abruption. Finally, while we identified significant predictors of adverse outcomes, the observational nature of our study precludes causal inferences. Furthermore, the limited number of similar studies makes it challenging to engage in a comprehensive discussion.

## Conclusion and implication

The prevalence of adverse perinatal outcome of pregnancy complicated with placental abruption is this study was high compared to the study done in similar setting. The degree of placental abruption and gestational age at diagnosis were significant associates of adverse perinatal outcomes. The study highlights the critical need for patient-centered counseling on antenatal bleeding to encourage early healthcare-seeking behavior, close follow up for those undergoing expectant management and the early detection and management of placental abruption to improve perinatal outcomes.

Our research indicates that placental abruption is associated with a higher risk of low birth weight, preterm delivery, neonatal resuscitation, and NICU admission. This suggests a possible link between abruption and physiological underdevelopment. Additionally, patients under expectant management may experience hypoxia and a gradual worsening of the condition. It is important to have the recommended antenatal care contact for improving the overall maternal and fetal outcomes.

## Data Availability

The raw data supporting the conclusions of this article will be made available by the authors, without undue reservation.
